# Natural and non-natural amino-acid side-chain substitutions: affinity and diffraction studies of meditope–Fab complexes

**DOI:** 10.1107/S2053230X16016149

**Published:** 2016-10-24

**Authors:** Krzysztof P. Bzymek, Kendra A. Avery, Yuelong Ma, David A. Horne, John C. Williams

**Affiliations:** a Department of Molecular Medicine, Beckman Research Institute of City of Hope, 1710 Flower Street, Duarte, CA 91010, USA

**Keywords:** meditope, monoclonal antibody, cetuximab, unnatural amino acids, X-ray crystallography, surface plasmon resonance

## Abstract

Herein, multiple crystal structures of meditope peptide derivatives incorporating natural and unnatural amino acids bound to the cetuximab Fab domain are presented. The affinity of each derivative was determined by surface plasmon resonance and correlated to the atomic structure. Overall, it was observed that the hydrophobic residues in the meditope peptide, Phe3, Leu5 and Leu10, could accommodate a number of moderate substitutions, but these invariably reduced the overall affinity and half-life of the interaction. In one case, the substitution of Phe3 by histidine led to a change in the rotamer conformation, in which the imidazole ring flipped to a solvent-exposed position. Based on this observation, Phe3 was substituted by diphenylalanine and it was found that the phenyl rings in this variant mimic the superposition of the Phe3 and His3 structures, producing a moderate increase, of 1.4-fold, in the half-life of the complex. In addition, it was observed that substitution of Leu5 by tyrosine and glutamate strongly reduced the affinity, whereas the substitution of Leu5 by diphenyl­alanine moderately reduced the half-life (by approximately fivefold). Finally, it was observed that substitution of Arg8 and Arg9 by citrulline dramatically reduced the overall affinity, presumably owing to lost electrostatic interactions. Taken together, these studies provide insight into the meditope–cetuximab interaction at the atomic level.

## Abstract 2

The structure–affinity relationship of meditope–cetuximab complexes is investigated by measuring their affinity using surface plasmon resonance and determining their structures using X-ray crystallography.

## 1. Introduction   

**Figure 1:**
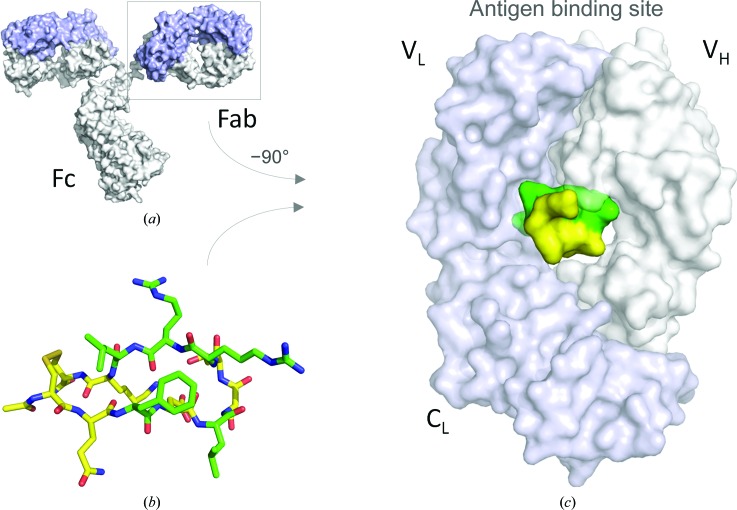
Meditope binding site. (*a*) Model of cetuximab IgG based on PDB entry 1igt (Harris *et al.*, 1997 ▸). The light chain is shown in light blue and the heavy chain in light gray. (*b*, *c*) The cavity in the Fab arm can accommodate the meditope peptide (PDB entry 4gw1; Donaldson *et al.*, 2013 ▸). The residues that are under investigation in this report are highlighted in green.

Monoclonal antibodies (mAbs) are used clinically to treat a number of diseases, including cancer, arthritis and Crohn’s disease (Emmons & Hunsicker, 1987 ▸; Suzuki *et al.*, 2015 ▸; Weiner *et al.*, 2012 ▸). Owing to their specificity and favorable pharmacological properties, there is significant interest in adding new functionality (*e.g.* potent cytotoxins) to increase their efficacy and potency (Trail & Bianchi, 1999 ▸; Wu & Senter, 2005 ▸; Chari, 2008 ▸; Ducry & Stump, 2010 ▸). Multiple strategies have been developed to modify monoclonal antibodies, including chemical conjugation (*e.g.* the introduction of cysteine; Bhakta *et al.*, 2013 ▸) and protein engineering (*e.g.* the introduction of an FGE site; Rabuka *et al.*, 2012 ▸). Recently, we identified a cyclic, 12-amino-acid peptide that binds in a cavity formed by all four IgG domains of the cetuximab Fab (Fig. 1 ▸; Donaldson *et al.*, 2013 ▸). Based on our observations, the peptide–Fab interaction does not affect antigen binding (Donaldson *et al.*, 2013 ▸). Furthermore, we have demonstrated that we could graft the binding site onto other Fabs, including trastuzumab, without affecting their antigen-binding properties (Donaldson *et al.*, 2013 ▸). Owing to the location of the binding site within the Fab, we have termed the peptide a meditope (*medi*-, middle, + -*tope*, place). To exploit this interaction as a potential ‘receptor’ within the Fab to rapidly add functionality to cetuximab and grafted mAbs without the need for covalent modification of the antibody, we have created a number of meditope variants, solved their structures and measured their affinity by surface plasmon resonance (SPR). In a previous manuscript, we focused on different cyclization strategies of the meditope peptide (Bzymek *et al.*, 2016 ▸). In this report, we focus on the modification of side chains that participate in the meditope–Fab interaction.

## 2. Materials and methods   

All reagents were obtained from Sigma or Fisher. Peptides were synthesized at the Synthetic and Biopolymer Chemistry Core, City of Hope, Duarte, California, USA or by CS Bio Co., Menlo Park, California, USA. The Leu5Tyr variant was biosynthesized as described elsewhere (Donaldson *et al.*, 2013 ▸). Fab purification, crystallization and structure solution were conducted as described previously (Bzymek *et al.*, 2016 ▸). Briefly, for each complex, the Fab was mixed with excess peptide (1:10–1:15 molar ratio of Fab:peptide). The precipitant solution was composed of 0.1 *M* Na_2_HPO_4_, 0.1 *M* citric acid, 0.4–0.5 *M* K_2_HPO_4_, 1.6–1.8 *M* NaH_2_PO_4_. Crystals were grown by vapour diffusion using the hanging-drop method at 20°C by mixing 1 µl protein complex solution with 1 µl precipitant solution. X-ray diffraction data were collected on a Rigaku MicroMax-007 HF with an R-AXIS IV^++^ detector (at a wavelength of 1.5418 Å) or on SSRL beamline 12-2 (L5Q; PDB entry 5th2; at a wavelength of 1.0000 Å). SPR experiments were performed on a GE Biacore T100 instrument (GE Healthcare). Briefly, cetuximab IgG was immobilized on a CM5 chip using amine-coupling chemistry at a density suitable for kinetics experiments with peptide analytes (∼5000 RU). Peptides were dissolved in 0.5 ml water and dialyzed with two changes against 500 ml water to remove excess TFA. Quantification of peptide concentration was performed as described previously (Bzymek *et al.*, 2016 ▸).

Analytes were passed over the chip at 30 µl min^−1^ in HBS-EP+ [10 m*M* HEPES pH 7.4, 150 m*M* NaCl, 3 m*M* EDTA, 0.05%(*v*/*v*) surfactant P20], which was used as both running and regeneration buffer. Data were processed with the *Biacore T100 Evaluation* software v.2.0.1. Data-collection and model statistics are presented in Table 1 ▸. All structures of cetuximab Fab–meditope complexes have been deposited in the RCSB PDB (http://www.rcsb.org) with the following accession codes: CQYDLSTRRLKC (F3Y), 5t1m; CQHDLSTRRLKC (F3H), 5euk; GQQDLSTRRLKG (F3Q), 5i2i; GQ(2-Br-F)DLSTRRLKG [F3(2-BrF)], 5itf; GQ(3-Br-F)­DLSTRRLKG [F3(3-BrF)], 5ir1; GQ(4-Br-F)DLSTRRL­KG [F3(4-BrF)], 5iop; CQA(Ph)_2_DLSTRRLKC [F3A(Ph)_2_], 5t1l; CQFDYSTRRLKC (L5Y), 5f88; CQFDESTRRLKC (L5E), 5etu; CQFDQSTRRLKC (L5Q), 5th2; CQFDA(Ph)_2_STRR­LKC [L5A(Ph)_2_], 5t1k; GQFDLST(Cit)RLKG (R8Cit), 5ivz; GQFDLSTR(Cit)LKG (R9Cit), 5iv2; CQFDLSTRRQKC (L10Q), 5ff6.

## 3. Results and discussion   

To exploit the Fab–meditope interaction for drug delivery, imaging and enhanced internalization, we sought to modulate the affinity of the interaction through the modification of meditope side chains. Moreover, because the meditope peptide consists of only 12 residues, it is straightforward to incorporate non-natural amino acids using routine solid-state peptide-synthetic methods. In our initial discovery and characterization of the interaction, mutagenesis of phenylalanine at position 3, leucine at position 5 and arginine at position 8 to alanine significantly reduced the affinity of the interaction (Donaldson *et al.*, 2013 ▸). Here, we further characterize these sites as well as Arg9 and Leu10. Of the remaining positions, Asp4, Ser6 and Thr7 form a class 3 β-hairpin (Milner-White & Poet, 1986 ▸) and are likely to be important for tertiary structure, while positions 2 and 11 are solvent-exposed and are associated with high *B* factors. Thus, we did not include these residues in this analysis. We were specifically interested in increasing the half-life of the interaction, which is independent of peptide concentration (note that the dissociation constants, *K*_d_, for some variants were difficult to calculate given the difficulty in determining the concentration of some of the meditope variants and we estimate that there is a 38% error in *K*_d_; see Bzymek *et al.*, 2016 ▸). Of note, there are two meditope–Fab complexes in the asymmetric unit and thus we observe each interaction twice.

### 3.1. Substitutions at position 3   

**Figure 2:**
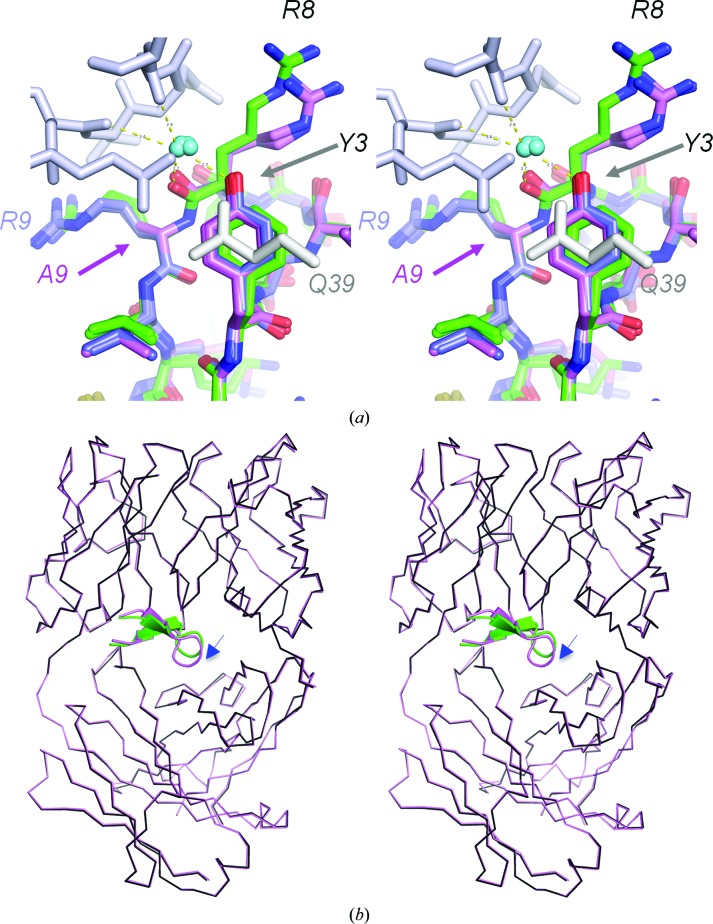
Tyrosine at position 3 affects Arg8 (stereoviews). (*a*) Superposition of cQYN (pink C atoms) and F3Y, where the alanine at position 9 (A9) is substituted by Arg (R9) (light blue C atoms), superimposed on the cQFD meditope (green C atoms). The presence of a hydroxyl from Tyr3 (Y3) sterically occludes the Arg8 side chain, resulting in the loss of a hydrogen bond to the backbone of Gln111 in the heavy chain. The hydroxyl group of Y3, however, leads to the coordination of a water molecule. (*b*) Superposition of the Fab (cQFD in black and cQYN in pink) shows that the hydroxyl substitution leads to a slight reorientation of the meditope with respect to the Fab.

Firstly, we focused on substitutions at position 3. In the original cQFD meditope, we observed that the phenylalanine ring in position 3 stacks against the amide group of Gln39 in the heavy chain (Gln39 HC), potentially contributing to the overall binding affinity of the meditope through π-stacking (James *et al.*, 2011 ▸). We also observed the same rotamer conformation in a meditope variant, cQYN, bound to the cetuximab Fab (Donaldson *et al.*, 2013 ▸). However, the cQYN peptide bound to the Fab with a much lower affinity (*K*_d_ = 3.5 µ*M*; Donaldson *et al.*, 2013 ▸). Superposition of the backbone peptide residues of the cQFD and cQYN variants (but not the Fab chains) indicated that the hydroxyl group of the tyrosine of cQYN sterically interferes with the position of the side chain of Arg8, breaking an electrostatic interaction between the guanidinium group and Gln111 HC observed in the original cQFD–Fab structure (Donaldson *et al.*, 2013 ▸). While the loss of this electrostatic bond could account for the difference in affinity, substitution of the tyrosine led to the coordination of a water molecule between the hydroxyl and the backbone carbonyl O atom of Arg39 and Gly42 of the light chain and induced an extended water network between the meditope and the Fab (Fig. 2 ▸ *a*). This coordination led to a slight reorientation of the meditope with respect to the Fab (Fig. 2 ▸ *b*). The r.m.s.d. between the Fabs is 0.53 Å calculated over 12 C^α^ atoms.

**Figure 3:**
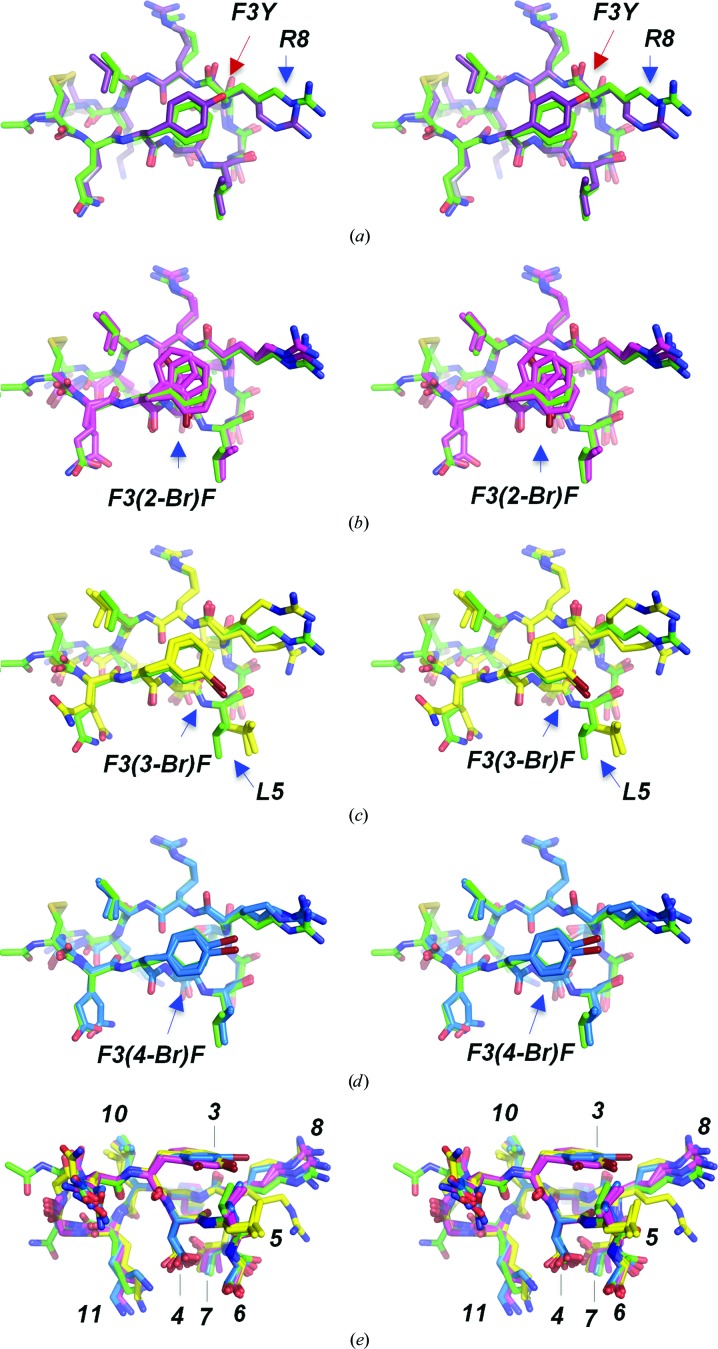
Substitutions of the phenylalanine at position 3 with brominated phenylalanine analogues, shown in stereo and superimposed on the cQFD meditope (green C atoms). (*a*) Viewed from the top, tyrosine (purple C atoms) at position 3 blocks the extension of the side chain of Arg8 (R8). (*b*) Substitution with 2-bromophenylalanine (2-BrF; magenta C atoms) does not affect the positioning of R8; however, there are multiple conformation of the 2-BrF side chain. (*c*) Substitution with 3-bromophenylalanine (3-BrF; yellow C atoms) affects R8; however, it also produces a conformational change in Leu5 (L5). (*d*) Substitution with 4-bromophenylalanine (4-BrF; blue C atoms) slightly perturbs R8. (*e*) Side view with each variant superimposed on the cQFD meditope.

In addition to the substitution of Phe3 by tyrosine, Arg9 was also substituted with an alanine in the original cQYN meditope. To determine whether the loss of affinity of the cQYN meditope compared with the cQFD meditope was owing to this substitution, we constructed a Phe3Tyr variant of the cQFD peptide. The affinity of this meditope variant, *K*_d_ = 1.5 m*M*, was slightly better than that of the original cQYN (*K*_d_ = 3.5 m*M*; Table 2 ▸), but remained weaker than that of the original cQFD peptide. The structure of this meditope variant bound to the Fab was very similar to that of the cQYN meditope (r.m.s.d. of 0.22 Å calculated over 12 C^α^ atoms). As before, the hydroxyl group of Tyr3 sterically blocked the Arg8 side chain from making the backbone hydrogen bond, coordinated a water molecule and was slightly reoriented compared with the original cQFD meditope (Fig. 3 ▸ *a*). Moreover, the side chain of Arg9 mimicked the same rotamer conformation as found in the cQFD meditope. Thus, the lower affinity is likely to be owing to loss of the electrostatic interaction between the guanidinium of Arg8 and the backbone carbonyl of Gln111 HC caused by the hydroxyl group of Tyr3.

Next, we tested whether the incorporation of a halogen in the phenyl group could afford a halogen–hydrogen bond between the meditope and Tyr87 LC that could increase the affinity (Figs. 3 ▸; Voth *et al.*, 2009 ▸). The structure with bromine at the *ortho* (2) position indicated that the aromatic ring lies in the same plane as the phenyl group of Phe3, while Arg8 was able to maintain the backbone hydrogen bond (Fig. 3 ▸ *b*). We observed electron density suggesting two conformations of 2-bromophenylalanine in one of the two meditopes in the asymmetric unit, with one of the Br atoms 3.2 Å from the OH of Tyr87 in the light chain (Tyr87 LC; Supplementary Fig. S2). This inter­action did not translate to higher affinity; in fact, bromine at the *ortho* position (2) of the phenyl ring significantly reduced the affinity compared with the original meditope (*K*_d_ = 1.5 m*M*; Table 2 ▸), but was an improvement over the original meditope cyclized by a diglycine linker (*K*_d_ = 5 m*M*; Bzymek *et al.*, 2016 ▸).

Next, we placed bromine at the *meta* position (3), again to test whether it could interact with the hydroxyl of Tyr87. The structure revealed that the bromine points away from Tyr87, forcing the side chain of Leu5 in the meditope to adopt a different conformation which is not observed in any of the complexes with other meditope variants (Fig. 3 ▸ *c*). This re­positioning of the Leu5 side chain also produced a shift of the entire meditope with respect to the Fab. Moreover, greater disorder was observed in the side chain of Arg8 of one of the meditopes in the asymmetric unit (*B* factor of 64.8 Å^2^ compared with 51.2 Å^2^). The binding affinity was also reduced (Table 2 ▸).

Finally, we also produced and characterized a 4-bromophenylalanine variant of the phenylalanine at position 3. Surprisingly, in one meditope–Fab complex in the asymmetric unit the Arg8 side chain is in an extended conformation similar to that in the original meditope (Fig. 3 ▸ *d*). The guanidinium in the 4-bromophenylalanine variant makes similar hydrogen bonds as in the original meditope (the bond distances NH1/NH2⋯O=C are 3.1/2.9 Å and 2.6–2.8/2.8–2.9 Å, respectively). In the other complex, the Arg8 side chain is not extended, placing the guanidinium group distant from the carbonyl (bond distances NH1/NH2⋯O=C of 3.3/4.0 Å). Of note, the coordinated water that was observed in the tyrosine variants is not observed in either complex of the 4-bromophenylalanine variant. This loss could be related to an unfavorable geometry owing to a slightly longer bond [C—Br *versus* C—O(H)]. On the other hand, the bromine in 4-bromophenylalanine is 3.7 and 3.9 Å from the hydroxyl of Tyr94 HC, forming a weak halogen–hydrogen bond (Voth *et al.*, 2009 ▸; Fig. 3 ▸ *d*). While the contribution of each of these potential explanations is unclear, the bromine is positioned such that it does not interfere with the side chain of Arg8. Based on these observations, we anticipated that the affinity of this meditope variant would be higher; however, it was in fact weaker (*K*_d_ = 2.8 µ*M* for the disulfide-bonded peptide) compared with the Phe3Tyr meditope (*K*_d_ = 1.2 µ*M*; Table 2 ▸). Of note, the structure was solved with a diglycine-linked peptide, which also showed reduced affinity for cetuximab (*K*_d_ = 29 µ*M* *versus* 5 µ*M* for the respective diglycine-linked meditope (Table 2 ▸; Bzymek *et al.*, 2016 ▸).

To test whether other side chains could be substituted at position 3 to form additional bonds to the Fab and/or through the water network, we used *PyMOL* (Schrödinger) to identify side chains and different rotamers and to replace the phenyl­alanine with a glutamine and a histidine. The histidine side chain is aromatic but is capable of acting as a proton acceptor or donor (depending on the pH). Also, the modelled rotamers placed the glutamine side chain within a suitable distance from several proton donors/acceptors for the formation of hydrogen bonds.

**Figure 4:**
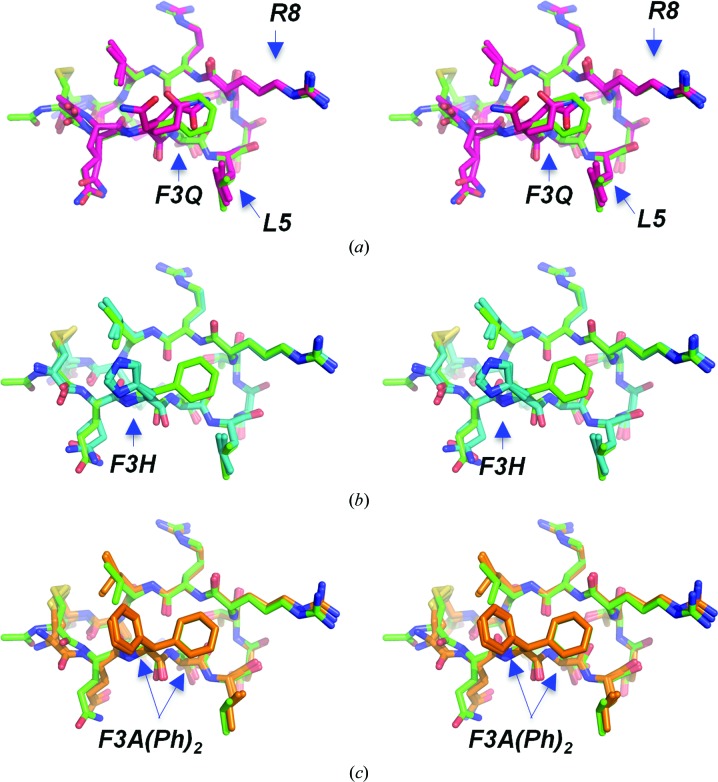
Substitutions of the phenylalanine at position 3, shown in stereo and superimposed on the cQFD meditope (green C atoms). (*a*) Substitution of phenylalanine with glutamine led to multiple side-chain rotamers (hot pink C atoms). (*b*) Substitution of phenylalanine with histidine led to a single conformation exposed to the solvent (cyan C atoms). (*c*) Based on these observations, we substituted phenylalanine with diphenylalanine (orange C atoms). One phenyl group of the diphenylalanine substitution superposed with the phenyl ring of the cQFD meditope. The other phenyl group superposed well with the imidazole ring of the histidine meditope variant.

While the crystal structure of the glutamine meditope variant shows significant disorder for the glutamine side chain (Fig. 4 ▸ *a*), the imidazole side chain of the histidine variant adopts a different rotamer from that of the original phenyl­alanine (Fig. 4 ▸ *b*). The imidazole ring is rotated ∼170° around the C^α^—C^β^ bond, losing the potential π-stacking with the highly conserved Gln–Gln interaction between the light and heavy chains. Rather, the imidazole makes weak interactions with the backbone carboxyl O atom of Ala100 LC (*d*_NE2⋯O=C_ of ∼3.1–3.2 Å) and its amide N atom (*d*_NH⋯ND1_ of 3.0–3.3 Å) (Fig. 4 ▸ *b* and Supplementary Fig. S3). We note that the pH of the crystallization buffer of 5.5 (which is close to the p*K*_a_ of the imidazole ring of histidine) should have afforded a mostly protonated form of histidine. We also note that at the pH value of 7.4 used for SPR experiments over 90% of histidine (assuming that the p*K*_a_ of a free histidine imidazole side chain is 6.0) would be in its unprotonated form, potentially weakening this interaction. SPR measurements indicate that the variants exhibited weaker binding to the Fab than the original meditope (Table 2 ▸).

Superposition of the F3H complex onto the cQFD complex, however, suggested that it may be possible to increase the affinity by introducing a β-branched, unnatural amino acid containing a phenyl group. Owing to its commercial availability, we incorporated diphenylalanine at position 3. The crystal structure of this variant indicated that the two phenyl rings mimic the phenyl and imidazole rings in the original and the F3H structures (Fig. 4 ▸ *c*), with no significant changes in the overall tertiary structure (r.m.s.d. of 0.6 Å calculated over 12 C^α^ atoms). Unlike the other substitutions at the F3 position, the affinity of this variant for cetuximab is similar to that of the original meditope (*K*_d_ = 0.24 µ*M* *versus* 0.17 µ*M*, respectively) with an off-rate that is approximately 40% slower. While the concentration of the peptides was difficult to determine owing to the low extinction coefficient (Bzymek *et al.*, 2016 ▸), off-rates are independent of concentration and thus comparison of the off-rates is more accurate. Given this, every substitution at position F3 significantly increased the off-rate (*e.g.* nearly tenfold) except for diphenylalanine. We speculate that the affinity could be further improved using a β-branched amino acid in which the exposed phenyl group is hydrophilic and capable of making hydrogen bonds to the Fab.

### 3.2. Substitutions at position 5   

**Figure 5:**
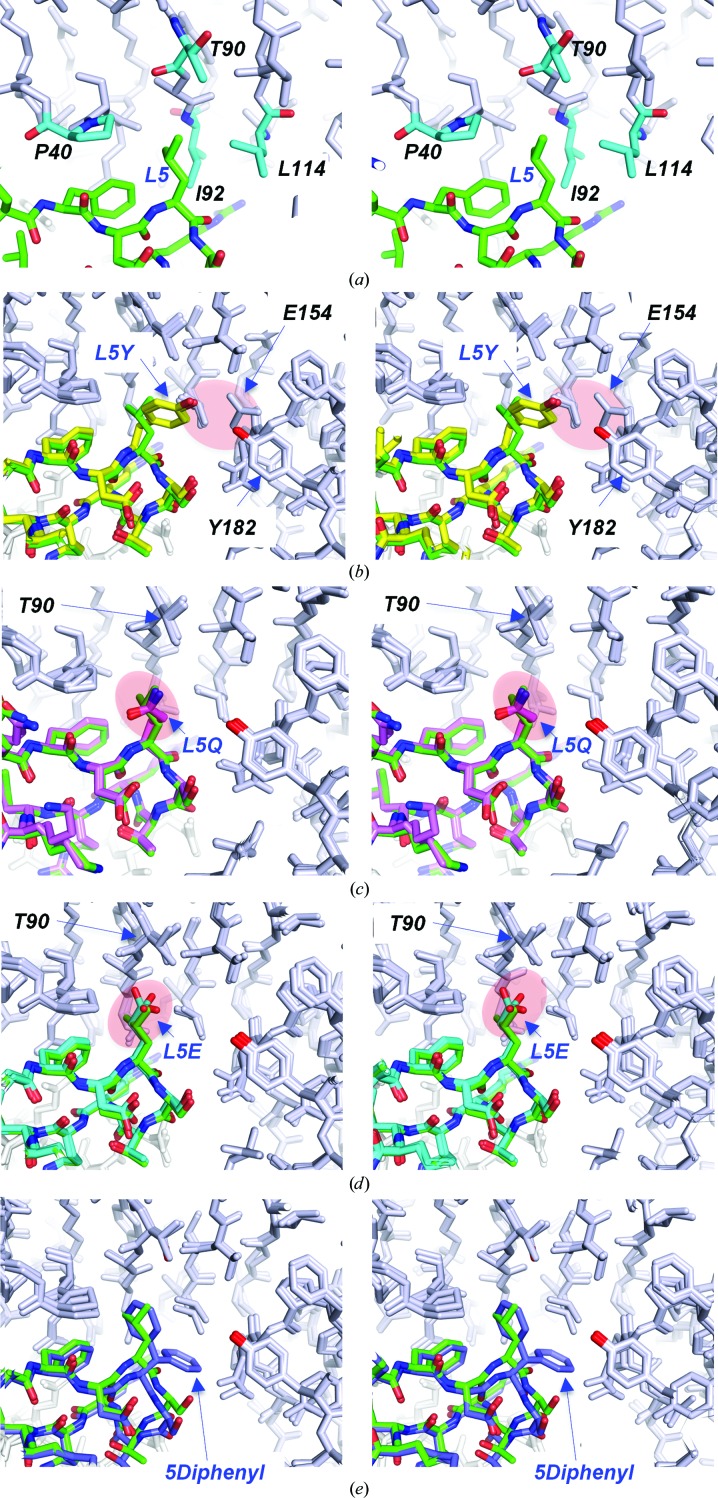
Substitutions of leucine at position 5, shown in stereo and superimposed on the cQFD meditope (green C atoms). (*a*) The leucine side chain resides in a hydrophobic pocket defined by Thr90 (T90), Ile92 (I92) and Leu114 (L114) of the Fab heavy chain and Pro40 (P40) of the Fab light chain. (*b*) Substitution of leucine with tyrosine in the meditope positions the hydroxyl group near the side chain of Glu154 (E154) and the hydroxyl group of Tyr182 (Y182). (*c*) The replacement of leucine with glutamine at position 5 was intended to create a hydrogen bond to the hydroxyl group of Y182 in the heavy chain or the hydroxyl of T90 in the light chain. However, the side chain points away from the Fab. (*d*) Substitution with glutamic acid resulted in positioning of the carboxylic acid in the hydrophobic pocket. The high *B* factors of the carboxylate suggest that the positioning of the side chain is adventitious. (*e*) Substitution at position 5 with diphenylalanine places one phenyl group at the same position as the leucine side chain. The other phenyl group extends further into the meditope cavity that is lined with the hydrophobic residues.

Next, we turned our attention to leucine at position 5 in the meditope peptide. Leu5 resides in a relatively hydrophobic pocket lined with Pro41 HC, Thr90 HC, Ile92 HC and Leu114 HC (Fig. 5 ▸ *a*). In an attempt to retain hydrophobic interactions and create additional interactions through hydrogen bonds, we substituted the leucine with a tyrosine (Fig. 5 ▸ *b*). The hydroxyl group forms a weak hydrogen bond to the hydroxyl group of Tyr182 HC (3.6/3.8 Å). In addition, a rotamer of Glu154 HC observed in one of the complexes in the asymmetric unit forms an electrostatic bond to the tyrosine hydroxyl (*d*_OH⋯OE2_ = 3.4 Å). The same OE2 of Glu154 HC is within 2.6 Å of OG1 of Thr116 HC. The other rotamer of Glu154 is at a distance of 2.3 Å from OG of Ser6 of the meditope compared with the respective OG of Ser6 of the NCS-related meditope (2.9 Å). Despite an additional hydrogen-bonding network, the affinity of this mutant, *K*_d_ > 50 µ*M*, is over two orders of magnitude weaker compared with that of the original meditope (Table 2 ▸).

In a second effort to create favorable hydrogen-bonding interactions, Leu5 was substituted with glutamine and, separately, glutamate. Based on the few available cetuximab structures at the time, we anticipated that the hydroxyl of Thr90 could be oriented to favor interactions with the carbonyl O atom of a glutamine or glutamic acid. We also noticed an extended water network in an apo structure solved in-house; upon binding of the L5E/Q meditope, the waters could facilitate interactions between the meditope and Tyr182. Substituting Leu5 with the polar but uncharged glutamine reduced the affinity ∼200-fold, despite the absence of direct interactions between the amide of glutamine and cetuximab Fab (Fig. 5 ▸ *c*). Substitution with glutamate produced a weak hydrogen bond between OE2 of Glu5 and the peptide NH of Ala91 HC (*d*_OE2⋯NH_ = 3.1–3.2 Å). However, unsurprisingly there are fewer hydrophobic interactions (Fig. 5 ▸ *c*). Unfavorable repulsive electrostatic forces between glutamate in position 5 and Glu154 HC are likely to be responsible for the low affinity and the fast off-rate, which is ∼30 times that of the original meditope. The lack of productive interactions with the Fab resulted in a drastic ∼760-fold drop in affinity compared with the original meditope. In contrast, the uncharged glutamine variant has an off-rate that is only approximately nine times faster that of the original meditope.

Finally, we substituted Leu5 with diphenylalanine in an effort to increase hydrophobic interaction between the meditope and Fab. The structure of the diphenylalanine substitution shows that one phenyl ring is in a similar position to the leucine side chain, while the other phenyl group contacts Ile92, Leu114 and Pro155. There is a slight shift in the backbone residues to accommodate the bulky diphenyl­alanine. The affinity for this mutant was slightly reduced compared with the original meditope: *K*_d_ = 0.53 µ*M*. However, it was not as severe as the other substitutions, and the off-rate increased approximately fivefold compared with the original meditope.

### 3.3. Substitutions at positions 8 and 9   

**Figure 6:**
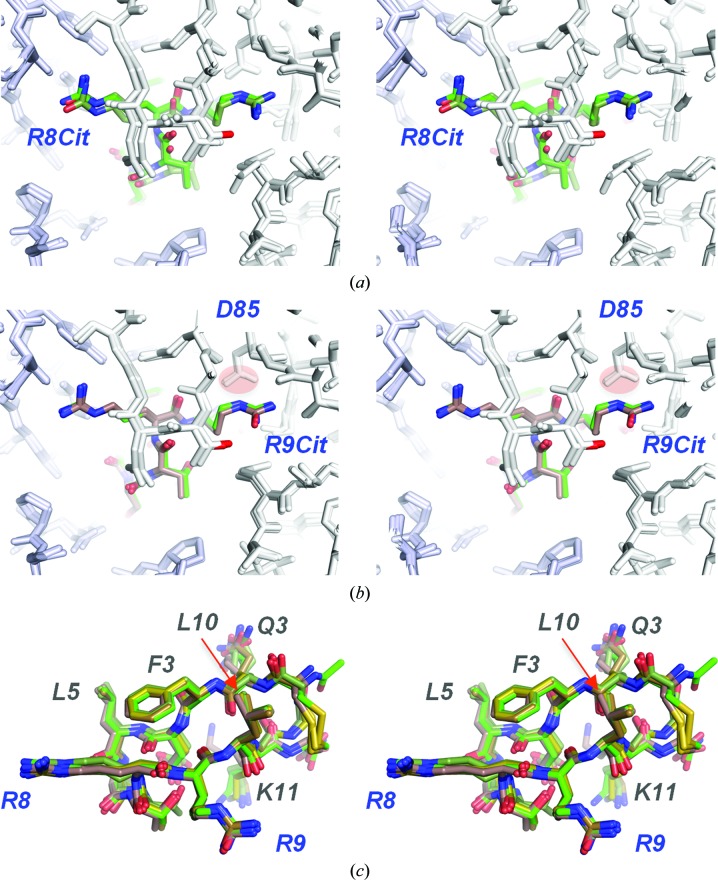
Citrulline substitutions, shown in stereo and superimposed on the cQFD meditope. The substitution of arginine with citrulline at either (*a*) position 8 or (*b*) position 9 gave structures that were indistinguishable from that of the original meditope (*c*).

As arginine side chains carry a positive charge at physiological pH, we tested the effect of substitution of the arginines with noncharged amino acids on binding to cetuximab Fab. Changes in the structure were minimal compared with the original diglycine-linked meditope (r.m.s.d.s of 0.131 and 0.125 Å for Arg8Cit and Arg9Cit, respectively, calculated over 12 C^α^ atoms; Fig. 6 ▸). Similar to the Arg8Ala modification, Arg8Cit showed reduced affinity for the meditope (*K*_d_ > 50 µ*M* *versus* *K*_d_ = 5 µ*M* for the arginine diglycine-linked peptide; Bzymek *et al.*, 2016 ▸). Beyond a difference in charge, the substitution of the amide for the guanidinium group also reduces the number of hydrogen bonds. There is one hydrogen bond between NH_2_ (the amide O atom in citrulline) and O=C of Gln111 HC (Donaldson *et al.*, 2013 ▸). Similarly, substitution of Arg9 with citrulline resulted in slightly weaker binding with *K*_d_ > 8 µ*M* (*K*_d_ = 5 µ*M* for the arginine diglycine-linked peptide; Bzymek *et al.*, 2016 ▸); the loss of affinity can be at least in part attributed to the loss of positive charge upon the substitution. The guanidinium group of Arg9 makes contacts with the carboxyl group of Asp85 from the light chain (Donaldson *et al.*, 2013 ▸). Thus, the charge from the guanidinium groups plays an important role in the overall affinity.

### 3.4. Substitutions at position 10   

**Figure 7:**
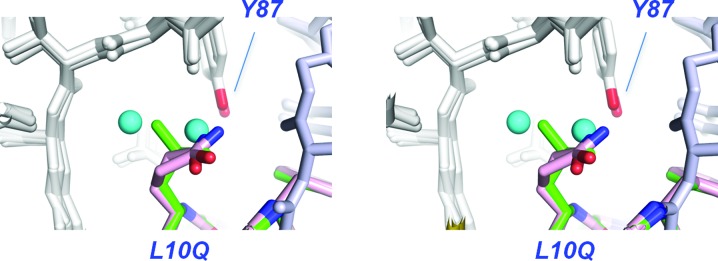
Substitutions of leucine at position 10, shown in stereo and superimposed on the cQFD meditope. Leu10 packs against a shallow hydrophobic pocket. Glutamine was substituted for Leu10 in an effort to form a hydrogen bond to the hydroxyl group of Tyr87 adjacent to the hydrophobic pocket. The cyan spheres represent two water molecules present in the apo structure (PDB entry 1yy8).

**Figure 8:**
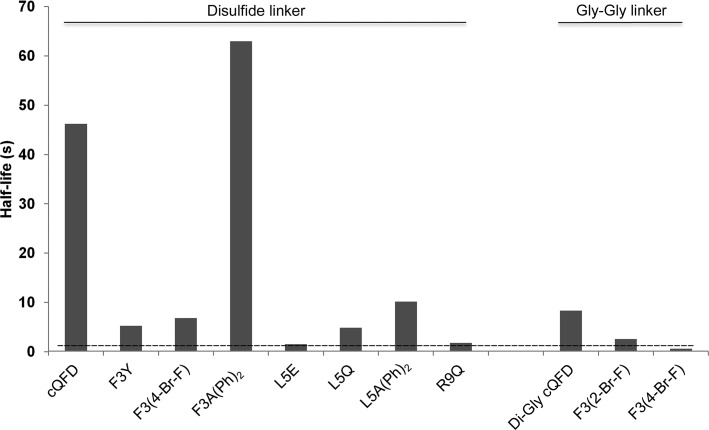
Half-lives of meditope variant–cetuximab interactions. While the on-rate of a bimolecular interaction is dependent on concentration, the off-rate is not. The half-life is related to the off-rate through *t* = ln(2)/*k*_d_. The dashed line represents the lower limit on the determination of *k*_d_ (0.5 s^−1^, corresponding to a 1.4 s half-life.) Note that several of the variants were cyclized through a diglycine linker, of which GQ(2-Br-F)DLSTRRLKG [F3(2-BrF)] and GQ(4-Br-F)DLSTRRLKG [F3(4-BrF)] allowed the determination of kinetic constants.

Leu10 resides in a relatively polar environment, and upon binding to the Fab it displaces three water molecules bound to the peptide backbone (Fig. 7 ▸). We substituted this side chain with the amide of glutamine in an effort to recapitulate the lost hydrogen-bonding interactions. While we observe that the amide O atom of Gln10 was 2.9 Å away from the hydroxyl of Tyr87, this seemingly productive interaction did not improve the affinity (*K*_d_ = 9.5 µ*M*) or the half-life of the complex (Table 2 ▸), showing a preference for hydrophobic side chains in this environment (Fig. 8 ▸).

## 4. Conclusion   

Our previous study focused on a cyclization strategy for the meditope peptide and its effect on the affinity for cetuximab Fab (Bzymek *et al.*, 2016 ▸). We determined that a disulfide-linked peptide exhibited the best kinetics in terms of the slowest off-rate (*k*_d_ = 0.015 s^−1^). Here, we present a detailed structure–binding analysis of multiple side-chain meditope variants of cetuximab Fab. We confirmed that hydrophobic interactions are preferred at positions 3, 5 and 10 of the meditope. Adding polar or charged side chains at those positions resulted in diminished binding, despite the presence of additional hydrogen bonds to the Fab framework. Despite the absence of a gain in binding affinity, the alternate rotamer observed for the histidine substitution at position 3 motivated the diphenylalanine substitution, which increased the lifetime of the interaction (Fig. 8 ▸). Attempts to reduce the charge of the residues in positions 8 and 9 resulted in weaker affinity. Interestingly, the change had a larger effect on Arg8 than Arg9, despite the fact that Arg9 forms a salt bridge with Asp85 of the cetuximab light chain. We are currently investigating substitutions on the antibody framework in an attempt to increase the affinity of the meditope–Fab interaction.

## Supplementary Material

PDB reference: cetuximab Fab, complex with CQYDLSTRRLKC meditope, 5t1m

PDB reference: with CQHDLSTRRLKC meditope, 5euk

PDB reference: with GQQDLSTRRLKG meditope, 5i2i

PDB reference: with GQ(2-Br-F)DLSTRRLKG meditope, 5itf

PDB reference: with GQ(3-Br-F)-DLSTRRLKG meditope, 5ir1

PDB reference: with GQ(4-Br-F)DLSTRRLKG meditope, 5iop

PDB reference: with CQA(Ph)_2_DLSTRRLKC meditope, 5t1l

PDB reference: with CQFDYSTRRLKC meditope, 5f88

PDB reference: with CQFDESTRRLKC meditope, 5etu

PDB reference: with CQFDQSTRRLKC meditope, 5th2

PDB reference: with CQFDA(Ph)_2_STRRLKC meditope, 5t1k

PDB reference: with GQFDLST(Cit)RLKG meditope, 5ivz

PDB reference: with GQFDLSTR(Cit)LKG meditope, 5iv2

PDB reference: with CQFDLSTRRQKC meditope, 5ff6

Supporting Information: Supplementary Figures S1-S3.. DOI: 10.1107/S2053230X16016149/rl5125sup1.pdf

## Figures and Tables

**Table:** Values in parentheses are for the highest resolution shell.

Meditope (PDB code)	F3H (5euk)	F3Y (5t1m)	F3Q (5i2i)	F3(2-BrF) (5itf)	F3(3-BrF) (5ir1)	F3(4-BrF) (5iop)	F3A(Ph)_2_ (5t1l)
Data collection							
Space group	*P*2_1_2_1_2_1_	*P*2_1_2_1_2_1_	*P*2_1_2_1_2_1_	*P*2_1_2_1_2_1_	*P*2_1_2_1_2_1_	*P*2_1_2_1_2_1_	*P*2_1_2_1_2_1_
*a*, *b*, *c* (Å)	63.93, 82.06, 212.09	63.93, 82.06, 212.09	63.97, 82.50, 211.88	64.01, 82.21, 211.90	64.04, 82.51, 211.54	64.05, 83.16, 212.26	64.02, 82.83, 212.10
α, β, γ (°)	90.0, 90.0, 90.0	90.0, 90.0, 90.0	90.0, 90.0, 90.0	90.0, 90.0, 90.0	90.0, 90.0, 90.0	90.0, 90.0, 90.0	90.0, 90.0, 90.0
Resolution (Å)	32.83–2.50 (2.56–2.50)	32.83–2.50 (2.56–2.50)	32.95–2.55 (2.62–2.55)	34.14–2.51 (2.58–2.51)	34.22–2.48 (2.55–2.48)	34.41–2.50 (2.56–2.50)	34.36–2.48 (2.54–2.48)
Wilson *B* factor (Å^2^)	33.5	31.1	35.2	38.1	31.3	32.6	31.5
*R*_meas_	0.126 (0.775)	0.066 (0.334)	0.074 (0.390)	0.046 (0.231)	0.046 (0.163)	0.082 (0.421)	0.065 (0.312)
CC_1/2_	0.993 (0.645)	0.999 (0.931)	0.998 (0.880)	0.999 (0.949)	0.999 (0.977)	0.998 (0.896)	0.999 (0.926)
〈*I*/σ(*I*)〉	11.3 (1.8)	24.7 (5.2)	17.8 (3.7)	25.2 (6.5)	30.1 (8.9)	20.5 (4.3)	24.9 (5.0)
Completeness (%)	99.2 (92.9)	97.6 (91.3)	99.0 (90.1)	98.5 (92.3)	99.4 (92.5)	99.2 (92.2)	99.4 (92.6)
Multiplicity	3.7 (3.0)	4.9 (4.5)	4.0 (3.7)	4.1 (3.5)	5.8 (4.1)	6.3 (4.9)	6.2 (4.3)
Refinement							
Resolution (Å)	2.50	2.53	2.55	2.51	2.48	2.50	2.48
No. of reflections	39421	37649	37030	38565	40287	39829	40642
*R*_work_/*R*_free_ (%)	17.1/23.4	15.3/21.6	16.0/20.5	18.4/22.3	16.3/21.5	17.7/22.6	15.8/20.5
No. of atoms							
Protein	6593	6635	6580	6551	6593	6536	6614
Meditope	200	204 (306)†	194	206	194	194	214
Water	410	475	333	369	506	414	548
*B* factors (Å^2^)							
Fab	26.2	27.9	33.2	39.6	30.1	29.9	21.7
Meditope	38.8	27.3 (46.8)†	40.2	52.6	53.4	40.6	28.3
Water	31.7	28.1	36.1	39.6	33.9	35.2	26.3
R.m.s.d.							
Bond lengths (Å)	0.008	0.007	0.007	0.003	0.008	0.004	0.008
Bond angles (°)	1.193	1.144	0.875	0.597	1.180	0.729	1.178
Ramachandran (favored/allowed/disallowed)	96.6/3.4/0.0	97.3/2.7/0.0	96.8/3.1/0.1	97.5/2.5/0.0	96.3/3.7/0.0	97.5/2.5/0.0	97.4/2.6/0.0

**Table:** 

Meditope (PDB code)	L5Y (5f88)	L5E (5etu)	L5Q (5th2)	L5A(Ph)_2_ (5t1k)	R8Cit (5ivz)	R9Cit (5iv2)	L10Q (5ff6)
Data collection							
Space group	*P*2_1_2_1_2_1_	*P*2_1_2_1_2_1_	*P*2_1_2_1_2_1_	*P*2_1_2_1_2_1_	*P*2_1_2_1_2_1_	*P*2_1_2_1_2_1_	*P*2_1_2_1_2_1_
*a*, *b*, *c* (Å)	64.24, 83.14, 211.94	64.38, 82.87, 213.00	64.19, 83.12, 212.56	64.28, 83.25, 212.30	64.34, 82.57, 212.05	64.14, 83.19, 212.46	64.08, 83.05, 212.67
α, β, γ (°)	90.0, 90.0, 90.0	90.0, 90.0, 90.0	90.0, 90.0, 90.0	90.0, 90.0, 90.0	90.0, 90.0, 90.0	90.0, 90.0, 90.0	90.0, 90.0, 90.0
Resolution (Å)	33.15–2.48 (2.55–2.48)	32.65–2.53 (2.60–2.53)	44.72–1.84 (1.89–1.84)	33.19–2.48 (2.54–2.48)	34.29–2.48 (2.54–2.48)	33.16–2.48 (2.55–2.48)	33.12–2.50 (2.56–2.50)
Wilson *B* factor (Å^2^)	29.1	32.8	33.2	31.4	26.5	24.6	217.1
*R*_meas_	0.053 (0.171)	0.096 (0.534)	0.060 (0.787)	0.060 (0.293)	0.039 (0135)	0.054 (0.186)	0.056 (0.185)
CC_1/2_	0.998 (0.980)	0.996 (0.790)	0.999 (0.677)	0.999 (0.917)	0.999 (0.987)	0.999 (0.969)	0.999 (0.969)
〈*I*/σ(*I*)〉	25.2 (9.2)	15.7 (3.0)	15.0 (2.0)	23.5 (4.7)	38.1 (11.4)	29.1 (8.3)	23.5 (7.3)
Completeness (%)	95.1 (72.3)	99.6 (98.1)	92.3 (94.6)	99.1 (90.3)	98.2 (91.1)	99.3 (93.2)	99.3 (92.6)
Multiplicity	5.2 (4.6)	3.9 (3.4)	3.5 (3.3)	4.6 (3.2)	6.2 (4.2)	5.8 (4.1)	4.8 (3.7)
Refinement							
Resolution (Å)	2.48	2.53	1.84	2.48	2.48	2.48	2.50
No. of reflections	39079	38822	91871	41103	40196	40876	39902
*R*_work_/*R*_free_ (%)	16.3/21.6	18.0/23.2	16.1/18.4	16.0/21.3	17.6/23.0	16.5/21.1	15.5/20.1
No. of atoms							
Protein	6566	6542	6731	6604	6571	6577	6614
Meditope	210	204	204	227	192	192	204
Water	512	389	850	518	537	617	532
*B* factors (Å^2^)							
Fab	27.8	28.8	30.6	20.7	20.3	22.5	28.5
Meditope	32.5	34.9	37.0	27.3	27.2	28.4	34.4
Water	32.5	32.8	44.0	25.7	26.4	30.5	33.6
R.m.s.d.							
Bond lengths (Å)	0.003	0.002	0.006	0.008	0.007	0.003	0.008
Bond angles (°)	0.664	0.683	0.873	1.149	0.916	0.644	1.180
Ramachandran (favored/allowed/disallowed)	97.6/2.4/0.0	97.0/2.9/0.1	98.4/1.6/0.0	97.5/2.5/0.0	96.7/3.3/0.0	96.9/3.1/0.0	96.8/3.2/0.0

† An additional meditope that was not bound to the meditope binding pocket in the F3H structure was identified and modeled in the asymmetric unit. The binding of a third copy of the peptide appeared to be adventitious and was facilitated by crystal contacts near CH of cetuximab Fab. The numbers in parentheses correspond to the number of atoms and the *B* factor calculated for all three peptides.

**Table 2:** Binding kinetics for meditope variants to cetuximab

Meditope		*k*_a_ (*M* ^−1^ s^−1^) × 10^4^	*k*_d_ (s^−1^)	*K*_d_ (µ*M*)
CQFDLSTRRLKC†	(cQFD)	8.8	0.015	0.17
GQFDLSTRRLKG†		1.7	0.083	5.0
CQYDLSTRRLKC	F3Y	8.5	0.132	1.5
CQQDLSTRRLKC	F3Q			>50‡
CQHDLSTRRLKC	F3H			>50‡
GQ(2-Br)FDLSTRRLKG	F3(2-Br)F	15	0.270	1.8
GQ(3-Br)FDLSTRRLKG	F3(3-Br)F			>5.4§
GQ(4-Br)FDLSTRRLKG	F3(4-Br)F			29‡
CQ(4-Br)FDLSTRRLKC	F3(4-Br)F disulfide	3.6	0.101	2.8
CQA(Ph)_2_DLSTRRLKC	F3A(Ph)_2_	4.5	0.011	0.24
CQFDESTRRLKC	L5E	0.34	0.444	130
CQFDQSTRRLKC	L5Q	0.46	0.148	30
CQFDYSTRRLKC	L5Y			>50‡
CQFDA(Ph)_2_STRRLKC	L5A(Ph)_2_	13	0.068	0.53
GQFDLST(Cit)RLKG	R8(Cit)			>50‡
GQFDLSTR(Cit)LKG	R9(Cit)			>8.0‡
CQFDLSTRRQKC	L10Q	4.1	0.390	9.5

† Data from Bzymek *et al.* (2016 ▸).

‡ Approximate value (*k*_a_ and/or *k*_d_ are outside the measurement range for the Biacore T100).

§ Affinity fit.
