# Supplementary material for: Natural and non-natural amino-acid side-chain substitutions: affinity and diffraction studies of meditope–Fab complexes
Source: Acta Crystallogr F Struct Biol Commun. 2016 Oct 24;72(Pt 11):820–30. doi: 10.1107/S2053230X16016149 (PMC5101583; doi:10.1107/S2053230X16016149)
Supplement: Supplementary file 1 [file f-72-00820-sup1.pdf]

STRUCTURAL BIOLOGY
COMMUNICATIONS

Volume 72 (2016)

Supporting information for article:

Natural and non-natural amino-acid side-chain substitutions: affinity and diffraction studies of meditope–Fab complexes

Krzysztof P. Bzymek, Kendra A. Avery, Yuelong Ma, David A. Horne and John C. Williams

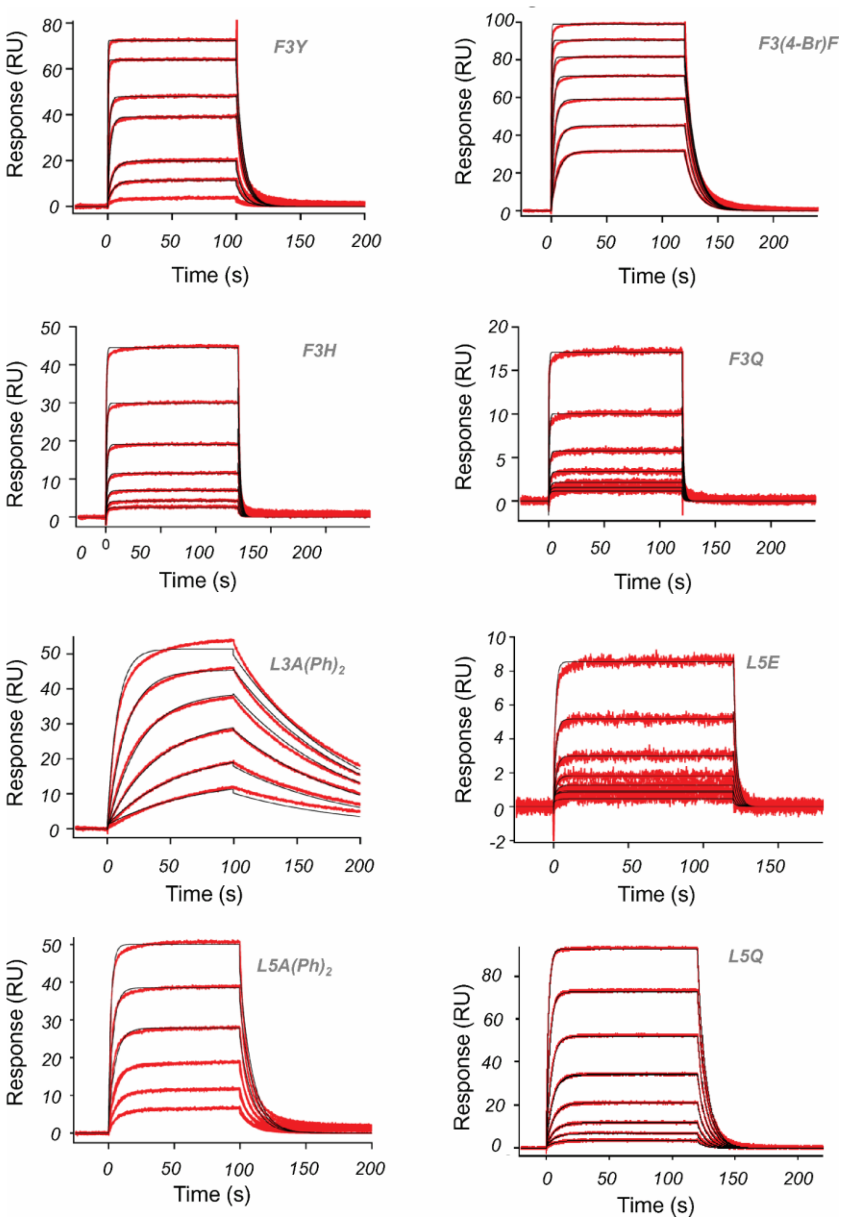
F3Y
F3(4-Br)F
F3H
F3Q
L3A(Ph)2
L5E
L5A(Ph)2
L5Q
Response (RU)
Time (s)

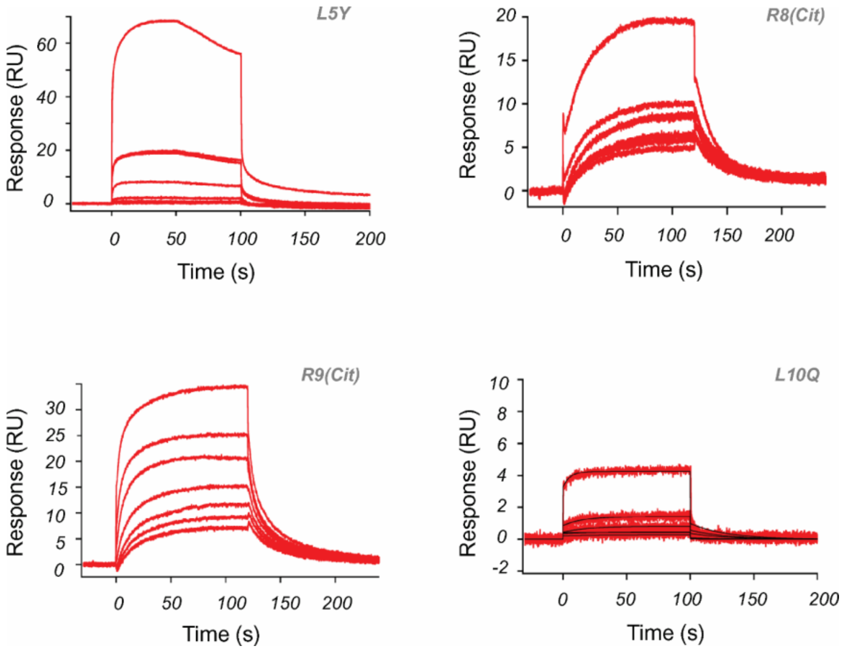

Figure S1 SPR sensograms of cetuximab – meditope variant interactions at 25°C.

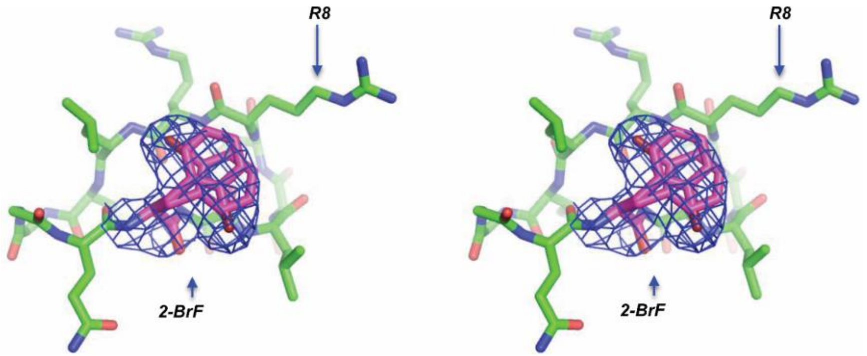

Figure S2 Composite omit map generated in Phenix (Adams *et al.*, 2010) for 2-bromophenylanine meditope variant and contoured at σ=1.0. Only a section of the map is shown (in blue).

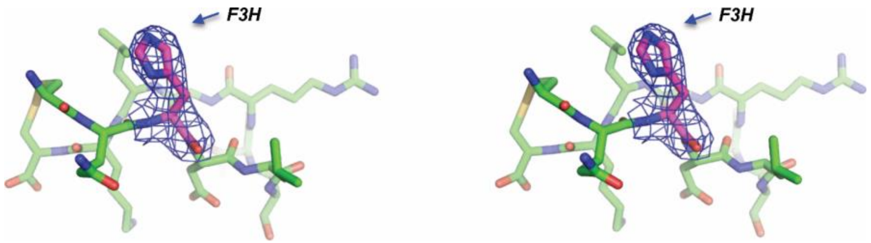

Figure S3 Composite omit map generated in Phenix (Adams *et al.*, 2010) for F3H meditope variant and contoured at σ=1.0. Only a section of the map is shown (in blue).
